# Antitumor Activity of Some Prenylated Xanthones

**DOI:** 10.3390/ph2020033

**Published:** 2009-08-11

**Authors:** Raquel A. P. Castanheiro, Artur M. S. Silva, Naïr A. N. Campos, Maria S. J. Nascimento, Madalena M. M. Pinto

**Affiliations:** 1 Centro de Química Medicinal da Universidade do Porto (CEQUIMED-UP), Faculdade de Farmácia, Universidade do Porto, Rua Aníbal Cunha 164, 4050-047 Porto, Portugal Email: raquelc@ff.up.pt (R.A.P.C.); 2 Departamento de Química & QOPNA, Universidade de Aveiro, Campus Universitário de Santiago, 3810-193 Aveiro, Portugal; 3 Serviço de Microbiologia, Faculdade de Farmácia, Universidade do Porto, Rua Aníbal Cunha 164, 4050-047 Porto, Portugal; 4 Serviço de Química Orgânica, Faculdade de Farmácia, Universidade do Porto, Rua Aníbal Cunha 164, 4050-047 Porto, Portugal

**Keywords:** xanthones, prenylation, dehydrogenation, antitumor activity, NMR spectroscopy

## Abstract

Pyranoxanthones **6**-**8** were obtained by dehydrogenation of the respective dihydropyranoxanthones **3**-**5** with DDQ in dry dioxane. Two prenylated xanthones **10**,**11** were obtained from the reaction of 1-hydroxyxanthone (**9**) with prenyl bromide in alkaline medium, or by condensation of xanthone **9** with isoprene in the presence of orthophosphoric acid. The structural elucidation of the two new compounds **6**,**11**, as well as an update of data for the already described prenylated derivatives **7**,**8**,**10** were accomplished by IR, UV, HRMS and NMR (^1^H, ^13^C, HSQC and HMBC) techniques. The effect of the prenylated xanthone derivatives on the *in vitro* growth of human tumor cell lines MCF-7 (breast adenocarcinoma) and NCI-H460 (non-small cell lung cancer) is also reported. Compounds **10** and **11** have been found to exhibit a moderate growth inhibitory activity against the MCF-7 cell line.

## 1. Introduction

**Figure 1:**
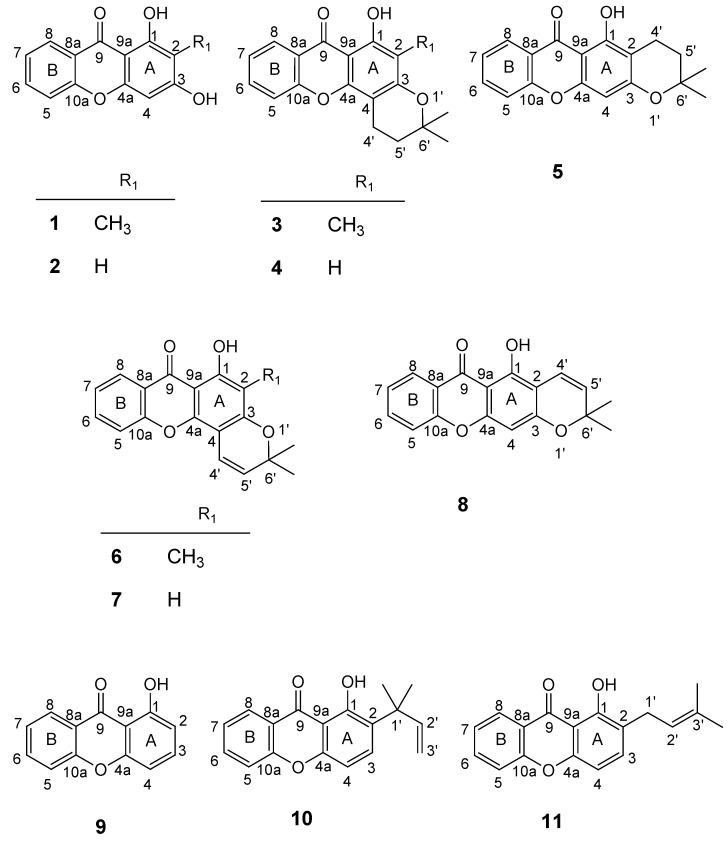
Structures of the xanthone building blocks **1**-**5**, **9** and prenylated derivatives **6-8**, **10** and **11** (the numbering used refers to the NMR assignments).

Many naturally occurring xanthones and their prenylated derivatives are found to exhibit significant biological and pharmacological properties, such as antibacterial, antifungal and antitumor activities and it can be inferred that the presence of prenyl groups can be associated with an improvement of potency and selectivity for some of these properties [1,2]. As a large number of biologically active xanthone derivatives with pyran and dihydropyran rings are commonly found in Nature, we were interested in obtaining this type of compounds to evaluate their antitumor activity. For this purpose, molecular modifications of the hit compounds, 1,3-dihydroxy-2-methylxanthone (**1**) and 1,3-dihydroxyxanthone (**2**) (Figure 1) were carried out [3]. 

Prenylation of xanthones **1** and **2** with prenyl bromide, followed by cyclisation of the respective monoprenylated products furnished dihydropyranoxanthones **3-5** [3] (Figure 1), which were evaluated for their effects on the *in vitro* growth of three human tumour cell lines (MCF-7, NCI-H460 and SF-268). These compounds were found to be more selective, showing their growth inhibitory effects only against the breast cancer MCF-7 cells when compared with their building blocks, respectively **1** and **2** [3].

The fact that naturally occurring pyranoxanthones are more active than dihydropyranoxanthones in many biological activity assays [1] has led us to resort to a rigidification strategy to improve the antitumor activity of the xanthone derivatives. Thus, unsaturation strategy was applied to the dihydropyran ring of dihydropyranoxanthones **3-5** to give pyranoxanthones **6-8**, respectively (Figure 1).

The second approach is to introduce the prenyl side chain to the xanthone nucleus, using a *C*-prenylation strategy. Thus, two *C*-prenylated derivatives, **10** and **11** were synthesized by prenylation of xanthone **9** (Figure 1). Though *C*-prenylated derivatives are not as common in nature as the *O*-prenylated analogues, they show very interesting properties [1]. Based on this observation, xanthone **9** (Figure 1) was submitted to a *C*-prenylation strategy to furnish compounds **10** and **11**.

The xanthone derivatives **6-8**, **10** and **11,** were then evaluated for their capacity to inhibit the *in vitro* growth of MCF-7 (breast adenocarcinoma) and NCI-H460 (non-small cell lung cancer) cells, and their effects were compared with those of their building blocks [3,4].

## 2. Results and Discussion

### 2.1. Synthesis of prenylated derivatives

**Scheme 1:**
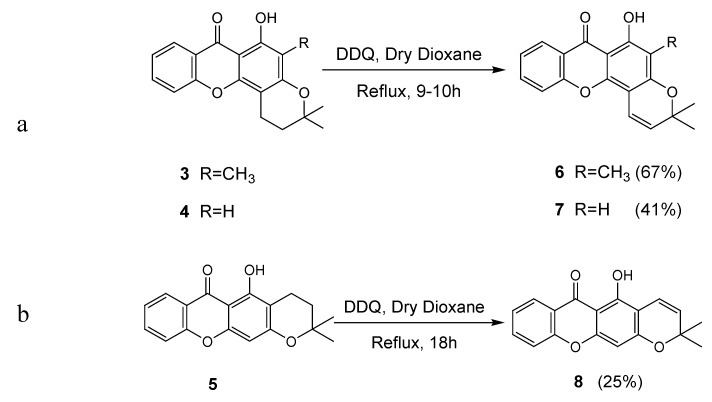
Synthesis of pyranoxanthones **6-8.** a). Synthesis of pyranoxanthones **6** and **7**; b). Synthesis of pyranoxanthone **8**.

Pyranoxanthones **6-8** were obtained by dehydrogenation of the respective dihydropyranoxanthones **3-5** with DDQ in refluxing dry dioxane [5]. While dihydropyranoxanthone **3** gave pyranoxanthone **6**, dihydropyranoxanthone **4** afforded pyranoxanthone **7** (Scheme 1a) and dihydropyranoxanthone **5** gave pyranoxanthone **8** (Scheme 1b) in 67, 41 and 25% yield, respectively.

**Scheme 2:**
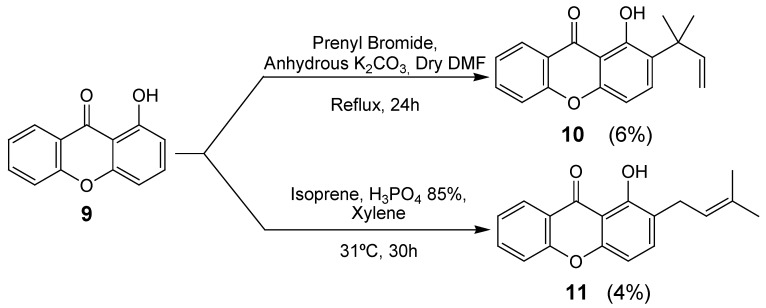
Synthesis of prenylated xanthones **10** and **11**.

Of these, pyranoxanthone **6**, containing a methyl group at C-2 was formed with the highest yield (67%). It can be also observed that the angular pyranoxanthone **7** was obtained in higher yield than the linear counterpart **8**. Prenylation of 1-hydroxyxanthone (**9**), either with prenyl bromide or isoprene, gave 1,1-dimethylallyl- or 3,3-dimethylallyl-derivatives **10** and **11**, respectively, in low yields and after long reaction times. Xanthone **10** was obtained by the reaction of 1-hydroxyxanthone (**9**) with prenyl bromide, in alkaline medium and refluxing *N,N*-dimethylformamide [6] (DMF) (Scheme 2).

Mechanistically, the formation of xanthone **10** by the described method, can be postulated to occur by prenylation at 1-OH of xanthone **9** with subsequent *ortho* Claisen rearrangement of the prenyl group to give the 1,1-dimethylallyl substituent on C-2 of the xanthonic scaffold [7].

The prenylated derivative **11** was obtained by condensation of 1-hydroxyxanthone (**9**) with isoprene, in the presence of catalytic amounts of orthophosphoric acid [8] (Scheme 2). The acid-catalysed condensation of isoprene with the phenol moiety of the xanthonic scaffold may be regarded as the chemical equivalent of the proposed biogenetic pathways [8].

### 2.2. Structural elucidation of the prenylated xanthones

The structures of compounds **6-8** and **10**, **11** were established by IR, UV, HRMS and NMR (^1^H-, ^13^C-, HSQC and HMBC) techniques, while the spectroscopic data of compounds **1-5** and **9** are in agreement with those reported in the literature [3,9,10,11,12]. Although the spectroscopic data of pyranoxanthones **7** and **8**, as well as of prenylated xanthone **10** have been previously described [7,13,14], here we provide an updated and complete structure elucidation of these compounds. 

The EI-HRMS of compound **6** gave the accurate molecular mass at 308.1049 and the corresponding molecular formula C_19_H_16_O_4_, indicating that there were two hydrogen atoms less than in its dihydropyranoxanthone precursor **3**. The ^1^H-NMR spectrum of compound **6** was very similar to that of compound **3**, except for the two doublets of the olefinic protons at *δ_H_* 5.62 (*J* = 10.0 Hz) and *δ_H_* 6.86 (*J* = 10.0 Hz), instead of the triplets of the protons of two methylene groups at *δ_H_* 1.88 (*J* = 6.8 Hz) and *δ_H_* 2.89 (*J* = 6.8 Hz) of the dihydropyran ring [3]. The protons of the geminal methyl groups of the pyran ring in compound **6** appeared as a singlet at *δ_H_* 1.50. The ^13^C-NMR spectrum of compound **6** was also similar to that of dihydropyranoxanthone **3** [3], except for the substitution of the two methylene carbons at *δ_C_* 16.4 and 31.7 with the two olefinic carbon signals at *δ_C_* 115.3 and *δ_C_* 126.8. 

In turn, the EI-HRMS of compound **7** indicated the accurate molecular mass at 294.0886, corresponding to the molecular formula C_18_H_14_O_4_. The ^1^H- and ^13^C-NMR spectra of compound **7** were very similar to those of compound **6**, except for the presence of a singlet of the aromatic proton at C-2 at *δ_H_* 6.28 instead of the singlet of the methyl group at *δ_H_* 2.12. As in compound **6**, the presence of the pyran ring in compound **7** was confirmed by the two doublets of the olefinic protons at *δ_H_* 5.62 (*J* = 10.0 Hz) and *δ_H_* 6.85 (*J* = 10.0 Hz) in the ^1^H-NMR spectrum which showed cross peaks with the olefinic carbons at *δ_C_* 127.2 and *δ_C_* 115.0, respectively in the HSQC spectrum. 

The EI-HRMS of compound **8** gave the accurate molecular mass at 294.0898 and the molecular formula C_18_H_14_O_4_. As expected, the ^1^H- and ^13^C-NMR spectra of compound **8** were similar to those of its dihydropyranoxanthone precursor **5** [3], except for the signals of the olefinic protons (*δ_H_* 6.74, *d*, *J* = 10.0 Hz and *δ_H_* 5.61, *d*, *J* = 10.0 Hz) and carbons (*δ_C_* 115.4 and *δ_C_* 127.6). 

Finally, the EI-HRMS of compounds **10** and **11** indicated their accurate molecular masses at 280.1099 and 280.1096, respectively, and thus, a molecular formula C_18_H_16_O_3_ for both compounds. This molecular formula confirmed the prenylation of xanthone **9**. In turn, the ^1^H-NMR spectra of compounds **10** and **11** showed, besides, the proton signals corresponding to the non substituted aromatic ring of the xanthone nucleus, the signals of another two *ortho* coupled aromatic protons (*δ_H_* 6.88, *d*, *J* = 8.8 Hz; *δ_H_* 7.64, *d*, *J* = 8.8 Hz and *δ_H_* 6.76, *d*, *J* = 8.4 Hz; *δ_H_* 7.46, *d*, *J* = 8.4 Hz) and 1-OH (*δ_H_* 13.47, *s* and *δ_H_* 12.56, *s*). The presence of these two *ortho* coupled aromatic protons indicated that the prenylation occurred at C-2. That the side chain of compound **10** was 2-methylbut-3-en-2-yl was confirmed by the signals of the protons of the vinyl group at *δ_H_* 5.07, *dd* (*J* = 17.0, 1.2 Hz), *δ_H_* 5.02, *dd* (*J* = 11.0, 1.2 Hz) and *δ_H_* 6.28, *dd* (*J* = 17.0, 11.0 Hz) and the methyl groups at *δ_H_* 1.55, *s*, respectively. This was corroborated by the correlation between the proton signal at *δ_H_* 6.28, *dd* (*J* = 17.0, 11.0 Hz, H-2’) and the carbon signal at *δ_C_* 128.9 (C-2). On the other hand, the 3-methylbut-2-enyl side chain of compound **11** was established by the presence of the signals of the allylic proton at *δ_H_* 5.33, *t* (*J* = 7.4 Hz), the methyl protons at *δ_H_* 1.76, *s* and *δ_H_* 1.81, *s* and the methylene protons at *δ_H_* 3.53, *d* (*J* = 7.4 Hz). The HMBC spectrum of compound **11** also showed the correlation between the signal of the methylene protons (*δ_H_* 3.53, *d*) and the signal of C-1 at *δ_C_* 160.0.

**Figure 2:**
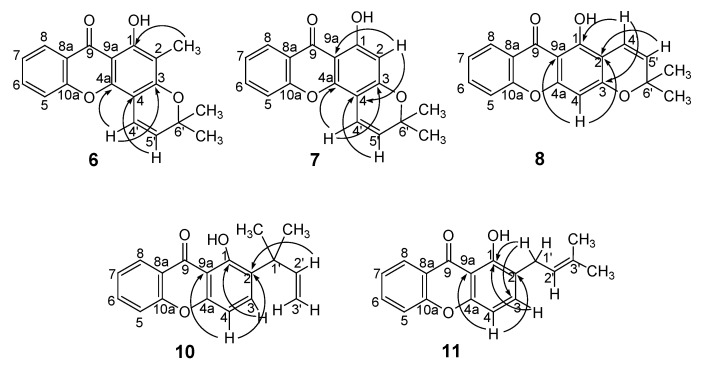
Main connectivities found in the HMBC of prenylated xanthones **6-8**, **10** and **11**.

### 2.3. Biological Activity studies

Though the number of compounds prepared was small, some basic structure-activity relationship trends can be observed. When the effects of the prenylated xanthones **6-8** on the growth of MCF-7 cells are compared with those of their respective xanthonic building blocks **3-5**, it was found that the presence of the unsaturation in the pyran ring was associated with a loss of inhibitory activity against MCF-7 (Table 1). It can be presumed that the lack of activity of compounds **6** and **8** could be a consequence of the rigidification of the dihydropyran ring. On the other hand, *C*-prenylation of the inactive xanthone **9** [4] was found to be associated with the growth of the inhibitory effect against MCF-7 of the prenylated derivatives **10** and **11** (Table 1). The introduction of the lipophilic prenyl group in C-2 of the xanthonic scaffold is probably the reason for the appearance of this activity for xanthones **10** and **11**. 

**Table 1:** Effect of xanthone derivatives **1-11** on the growth of human tumor cell lines.

Compound	GI_50_ (µM)		
	MCF-7	NCI-H460	SF-268
**1^a^**	21.9 ± 0.4	20.6 ± 0.9	33.4 ± 0.2
**2^a^**	50.8 ± 2.2	37.9 ± 2.9	61.4 ± 5.2
**3^a^**	18.4 ± 1.9	>160	>160
**4^a^**	>160	>160	>160
**5^a^**	88.6 ± 12.9	>160	>160
**6**	>150^b^	>150^b^	ND
**7**	>150	>150^b^	ND
**8**	>150^b^	>150^b^	ND
**9^a^**	>200	ND	ND
**10**	55^b^	>150^b^	ND
**11**	88^b^	ND	ND
Results are given in concentrations that were able to cause 50% of cell growth inhibition (GI_50_) after a continuous exposure of 48h and represent means of ±SEM of 3 independent experiments performed in duplicate and carry out independently. ^a^Results published elsewhere [3,4]. ^b^Results of one or two experiments performed in duplicate. Doxorubicin was used as positive control, GI_50_: MCF-7 = 42.8±8.2 nM; NCI-H460 = 94.0±8.7 nM; SF-268 = 93.0±7.0 nM. ND = not determined.			

## 3. Experimental

### 3.1. General

Purification of compounds were performed by flash chromatography using Merck silica gel 60 (0.040-0.063 mm) and preparative thin layer chromatography (TLC) using Merck silica gel 60 (GF_254_) plates. Reactions were monitored by TLC. Melting points were obtained in a Köfler microscope and are uncorrected. IR spectra were measured on an ATI Mattson Genesis series FTIR (software: WinFirst v. 2.10) spectrophotometer in KBr microplates (cm^-1^). UV spectra were taken in ethanol [15] and were recorded on a Varian CARY 100 spectrophotometer: *λ_max_* in nm (software: Cary Win UV v. 3.0). ^1^H and ^13^C NMR spectra were taken in CDCl_3_ at room temperature, on a Bruker Avance 300 instrument. Chemical shifts are expressed in *δ* (ppm) values relative to tetramethylsilane (TMS) as an internal reference. ^1^H-NMR spectra were measured at 300.13 MHz and assignment abbreviations are the following: singlet (*s*), doublet (*d*), triplet (*t*), quartet (*q*), multiplet (*m*), doublet of doublets (*dd*), and double doublet of doublets (*ddd*). ^13^C-NMR spectra were measured at 75.47 MHz. ^13^C-NMR assignments were made by 2D HSQC and HMBC experiments (long-range C, H coupling constants were optimized to 7 Hz). HRMS spectra were recorded as EI (electronic impact) mode on a VG Autospec M spectrometer (*m/z*) at *CACTI,* Vigo, Spain. Prenyl bromide, Isoprene and DDQ were purchased from Sigma Aldrich. Compounds **1-5** and **9** were obtained and characterized according to the described procedures [3,9,10,11,12]. The following materials were synthesized and purified by the described procedures.

### 3.2. General Procedure for the Synthesis of Pyranoxanthones ***6-8***

To a solution of dihydropyranoxanthones **3-5** (0.06 mmol) in dry dioxane (10 mL) was added DDQ (0.12 mmol) and the reaction mixture was refluxed (100ºC) for 9-18 h. After cooling, the precipitate was filtered off and the filtrate evaporated. The crude product was purified by preparative TLC (SiO_2_; hexane/EtOAc 95:5 or light petroleum/CHCl_3_ 5:5). Compounds **6, 7** [13] and **8** [14] were identified by their spectroscopic and analytical data.

*6-Hydroxy-3,3,5-trimethylpyrano[2,3-c]xanthen-7(3H)-one* (**6**): The compound was obtained (67%) as yellow solid; m.p. 196-198ºC (EtOH); *λ_max_* (ε): 327, 273, 250 (6821, 25216, 22253); (EtOH + NaOH): 426, 296, 217 (3056, 30278, 95370); (EtOH + AlCl_3_): 330, 275, 235 (6481, 24753, 17685); *ν_max_* (KBr): 3431, 2959, 2917, 1644, 1606, 1564, 1471, 1431, 1320, 1145, 1108, 742 cm^-1^; ^1^H-NMR: *δ* =13.22 (*s*, 1H, 1-OH), 8.25 (*dd*, 1H, *J* = 8.0, 1.6 Hz, 8-H), 7.70 (*ddd,* 1H, *J =* 8.4, 7.1, 1.6 Hz, 6-H), 7.44 (*d*, 1H, *J* = 8.4 Hz, 5-H), 7.36 (*dd*, 1H, *J =* 8.0, 7.1 Hz, 7-H), 6.86 (*d*, 1H, *J =* 10.0 Hz, 4’-H), 5.62 (*d*, 1H, *J=* 10.0 Hz, 5’-H), 2.12 (*s*, 3H, 2-CH_3_), 1.50 (*s*, 6H, 6’-CH_3_) ppm; ^13^C-NMR: *δ* =180.8 (C-9), 160.5 (C-1), 158.8 (C-3), 155.8 (C-10a), 149.8 (C-4a), 134.7 (C-6), 126.8 (C-5’), 125.9 (C-8), 123.8 (C-7), 120.6 (C-8a), 117.5 (C-5), 115.3 (C-4’), 107.8 (C-2), 103.1 (C-9a), 100.5 (C-4), 78.0 (C-6’), 28.4 (6’-*C*H_3_, 2C), 7.0 (2-*C*H_3_) ppm; EI-MS *m/z* (%): 308 (6, M^+.^), 293 (100), 267 (4), 149 (5), 137 (4), 121 (4), 109 (5), 95 (6), 81 (11), 69 (12); EI-HR-MS *m/z*: Anal. Calc. for C_19_H_16_O_4_: 308.1049; found: 308.1049.

*6-Hydroxy-3,3-dimethylpyrano[2,3-c]xanthen-7(3H)-one* (**7**): The compound was obtained (41%) as yellow crystals; m.p. 164-168ºC (Acetone); *λ_max_* (ε): 271, 244, 201 (8471, 7588, 5603); (EtOH + NaOH): 293, 216 (9103, 45176); (EtOH + AlCl_3_): 335, 285, 228, 201 (2838, 9412, 6941, 6147); *ν_max_* (KBr): 3432, 2956, 2921, 2853, 1650, 1596, 1465, 1279, 1142, 1102, 1073, 804, 749 cm^-1^; ^1^H-NMR: *δ* =12.97 (*s*, 1H, 1-OH), 8.25 (*d*, 1H, *J* = 7.8 Hz, 8-H), 7.72 (*dd,* 1H, *J =* 8.4, 7.4 Hz, 6-H), 7.46 (*d*, 1H, *J* = 8.4 Hz, 5-H), 7.38 (*dd*, 1H, *J =* 7.8, 7.4 Hz, 7-H), 6.85 (*d*, 1H, *J =* 10.0 Hz, 4’-H), 6.28 (*s*, 1H, 2-H), 5.62 (*d*, 1H, *J=* 10.0 Hz, 5’-H), 1.49 (*s*, 6H, 6’-CH_3_) ppm; ^13^C-NMR: *δ* =180.9 (C-9), 163.2 (C-1), 161.0 (C-4a), 155.8 (C-10a), 151.8 (C-3), 135.0 (C-6), 127.2 (C-5’), 125.9 (C-8), 124.1 (C-7), 120.6 (C-8a), 117.6 (C-5), 115.0 (C-4’), 103.8 (C-9a), 101.1 (C-4), 99.4 (C-2), 78.3 (C-6’), 28.3 (6’-*C*H_3_, 2C) ppm; EI-MS *m/z* (%): 294 (2, M^+.^), 279 (22), 183 (74), 181 (78), 171 (60), 169 (66), 163 (100), 149 (20), 145 (25), 117 (40), 115 (37), 104 (30), 103 (46), 91 (35), 90 (50), 89 (53), 77 (26); EI-HR-MS *m/z*: Anal. Calc. for C_18_H_14_O_4_: 294.0892; found: 294.0886.

*5-Hydroxy-2,2-dimethylpyrano[3,2-b]xanthen-6(2H)-one* (**8**): The compound was obtained (25%) as yellow crystals; m.p. 170-173ºC (Acetone); *λ_max_* (ε): 289, 237, 201 (17059, 14676, 11853); (EtOH + NaOH): 405, 309, 215 (1647, 14471, 88324); (EtOH + AlCl_3_): 293, 237, 201 (16471, 14765, 13324); *ν_max_* (KBr): 3410, 2963, 2921, 2855, 1646, 1609, 1567, 1453, 1301, 1212, 1139, 1081, 749 cm^-1^;^ 1^H-NMR: *δ* =13.17 (*s*, 1H, 1-OH), 8.24 (*dd*, 1H, *J* = 8.0, 1.6 Hz, 8-H), 7.70 (*ddd,* 1H, *J =* 8.6, 7.1, 1.6 Hz, 6-H), 7.43 (*d*, 1H, *J* = 8.6 Hz, 5-H), 7.37 (*dd*, 1H, *J =* 8.0, 7.1 Hz, 7-H), 6.74 (*d*, 1H, *J* = 10.0 Hz, 4’-H), 6.36 (*s*, 1H, 4-H), 5.61 (*d*, 1H, *J=* 10.0 Hz, 5’-H), 1.49 (*s*, 6H, 6’-CH_3_) ppm; ^13^C-NMR: *δ* =180.8 (C-9), 160.9 (C-3), 157.7 (C-1), 157.1 (C-4a), 155.9 (C-10a), 134.9 (C-6), 127.6 (C-5’), 125.8 (C-8), 124.0 (C-7), 120.5 (C-8a), 117.6 (C-5), 115.4 (C-4’), 107.1 (C-4), 104.6 (C-2), 103.8 (C-9a), 78.3 (C-6’), 28.4 (6’-*C*H_3_, 2C) ppm; EI-MS *m/z* (%): 294 (9, M^+.^), 279 (100), 69 (7); EI-HR-MS *m/z*: Anal. Calc. for C_18_H_14_O_4_: 294.0892; found: 294.0898.

### 3.3. Synthesis of Prenylated Xanthone ***10***

A mixture of 1-hydroxyxanthone **(9)** (0.10 g; 0.47 mmol), prenyl bromide (110 μL; 0.95 mmol) and anhydrous K_2_CO_3_ (0.22 g, 1.58 mmol) in dry DMF (7 mL), was refluxed at 150ºC for 24 h. After cooling, the solid was filtered and the solvent removed under reduced pressure, affording the crude product that was purified by flash chromatography (SiO_2_; Hexane/EtOAc 95:5) and by preparative TLC (SiO_2_; Hexane/CHCl_3_ 9:1). The product, *1-hydroxy-2-(2-methylbut-3-en-2-yl)-9H-xanthen-9-one* (**10**) [7] was obtained in 6% yield as yellow crystals, and identified by spectroscopic and analytical data; m.p. 99-102ºC (acetone); *λ_max_* (ε): 281, 258, 230, 203 (2511, 10196, 9453, 6858); (EtOH + NaOH): 426, 309, 216 (1641, 3815, 43184); (EtOH + AlCl_3_): 259, 231, 206 (7475, 9341, 6466); *ν_max_* (KBr): 3432, 2954, 2919, 2858, 1632, 1608, 1462, 1433, 1374, 1285, 1213, 1057, 752 cm^-1^;^ 1^H-NMR: *δ* =13.47 (*s*, 1H, 1-OH), 8.29 (*dd*, 1H, *J* = 8.0, 1.6 Hz, 8-H), 7.74 (*ddd,* 1H, *J =* 8.7, 7.0, 1.6 Hz, 6-H), 7.64 (*d*, 1H, *J* = 8.8 Hz, 3-H), 7.45 (*d*, 1H, *J* = 8.7 Hz, 5-H), 7.38 (*dd*, 1H, *J =* 8.0, 7.0 Hz, 7-H), 6.88 (*d*, 1H, *J* = 8.8 Hz, 4-H), 6.28 (*dd*, 1H, *J =* 17.0, 11.0 Hz, 2’-H), 5.07 (*dd*, 1H, *J=* 17.0, 1.2 Hz, 3’-H), 5.02 (*dd*, 1H, *J=* 11.0, 1.2 Hz, 3’-H), 1.55 (*s*, 6H, 1’-CH_3_) ppm; ^13^C-NMR: *δ* =182.9 (C-9), 160.5 (C-1), 156.1 (C-10a), 154.8 (C-4a), 147.0 (C-2’), 135.4 (C-6), 135.0 (C-3), 128.9 (C-2), 126.0 (C-8), 123.8 (C-7), 120.5 (C-8a), 117.7 (C-5), 110.6 (C-3’), 108.8 (C-9a), 105.6 (C-4), 40.3 (C-1’), 26.7 (1’-*C*H_3_, 2C) ppm; EI-MS *m/z* (%): 280 (20, M^+.^), 265 (100), 251 (17), 250 (16), 239 (16), 237 (20), 225 (35), 69 (11); EI-HR-MS *m/z*: Anal. Calc. for C_18_H_16_O_3_: 280.1100; found: 280.1099.

### 3.4. Synthesis of prenylated xanthone ***11***

A solution of isoprene (200 μL; 2.00 mmol) in xylene (1 mL) was added to a stirred mixture of 1-hydroxyxanthone (**9**, 0.20 mg; 0.96 mmol), orthophosphoric acid (85%, 1 mL) and xylene (4 mL), with constant stirring at 31ºC during 2 h. The mixture was stirred for a further 28 h and then neutralised with hydrogen carbonate solution (5%). The mixture thus obtained, was extracted with diethyl ether. The extract was washed with water, dried (Na_2_SO_4_) and the solvent evaporated under reduced pressure. The crude product thus obtained was purified by flash chromatography (SiO_2_; Hexane/EtOAc 98:2) and preparative TLC (SiO_2_; EP/Et_2_O 9:1). *1-Hydroxy-2-(3-methylbut-2-enyl)-9H-xanthen-9-one* (**11**) was identified by its spectroscopic and analytical data. Yield: 4%, as yellow crystals; m.p. 68-71ºC (acetone); *λ_max_* (ε): 368, 300, 257, 232, 203 (3240, 5526, 23689, 24109, 18794); (EtOH + NaOH): 416, 308, 265, 217 (4600, 9257, 16157, 49130); (EtOH + AlCl_3_): 445, 316, 275, 231, 205 (3394, 7798, 21837, 26452, 20084); *ν_max_* (KBr): 3448, 2963, 2917, 2853, 1642, 1604, 1472, 1369, 1279, 1227, 763 cm^-1^;^ 1^H-NMR: *δ* =12.56 (*s*, 1H, 1-OH), 8.29 (*dd*, 1H, *J* = 8.0, 1.6 Hz, 8-H), 7.76 (*ddd,* 1H, *J =* 8.4, 7.1, 1.6 Hz, 6-H), 7.51 (*d*, 1H, *J* = 8.4 Hz, 5-H), 7.46 (*d*, 1H, *J* = 8.4 Hz, 3-H), 7.40 (*dd*, 1H, *J =* 8.0, 7.1 Hz, 7-H), 6.76 (*d*, 1H, *J* = 8.4 Hz, 4-H), 5.33 (*t*, 1H, *J =* 7.4 Hz, 2’-H), 3.53 (*d*, 2H, *J=* 7.4 Hz, 1’-H), 1.81 and 1.76 (2*s*, 2´3H, 3’-CH_3_) ppm; ^13^C-NMR: *δ* =182.6 (C-9), 160.0 (C-1), 156.1 (C-10a), 153.4 (C-4a), 137.0 (C-3), 135.4 (C-6), 133.3 (C-3’), 126.0 (C-8), 124.0 (C-7), 121.7 (C-2’), 120.5 (C-8a), 119.3 (C-2), 117.9 (C-5), 110.0 (C-4), 108.9 (C-9a), 27.6 (C-1’), 25.8 and 17.9 (3’-*C*H_3_, 2C) ppm; EI-MS *m/z* (%): 280 (15, M^+.^), 265 (33), 225 (12), 149 (11), 137 (18), 121 (18), 109 (12), 107 (12), 95 (26), 81 (69), 69 (100); EI-HR-MS *m/z*: Anal. Calc. for C_18_H_16_O_3_: 280.1100; found: 280.1096.

### 3.5. Tumor cell growth assay

Stock solutions of compounds **6-8**, **10** and **11** and doxorubicin were prepared in DMSO (Sigma Chemical Co) and stored at –20 ºC. The frozen samples were freshly diluted with culture medium just prior the assays. Final concentrations of DMSO (0.25%) did not interfere with the growth of cell lines. 

The human tumor cell lines MCF-7 (breast adenocarcinoma) and NCI-H460 (non-small cell lung cancer) were used. Cells growing as monolayer, were routinely maintained in RPMI-1640 medium (Gibco BRL) supplemented with 5% heat-inactivated fetal bovine serum (Gibco BRL), 2 mM glutamine (Sigma Chemical Co.), penicillin 100 U/mL and 100 μg/mL streptomycin (Gibco BRL), at 37 ºC in an humidified atmosphere containing 5% CO_2_. The optimal plating density of each cell line, that ensure exponential growth throughout all the experimental period was respectively 1.5 × 10^5^ cells/ml to MCF-7 and 7.5 × 10^4^ cells/ml for NCI-H460. 

The effects of compounds on the growth of the human tumor cell lines were evaluated according to the procedure adopted by the National Cancer Institute (NCI, USA) for the “In vitro Anticancer Drug Discovery Screen” that uses the protein-binding dye sulforhodamine B (SRB) (Sigma Chemical Co.) to assess cell growth [16,17]. Briefly, exponentially growing cells were exposed for 48 h to five serial concentrations (1:2 or 1:3 dilution) of each compound, starting from a maximum concentration of 150 μM. Following this exposure period adherent cells were fixed in situ with 50% TCA, washed with distillate water and stained with 0.4% SRB solubilized in 1% acetic acid. The bound stain was solubilized and the absorbance was measured at 492 nm in a microplate reader (Bio-tek Instruments Inc., PowerWave XS, Winooski, USA). For each cell line a dose-response curve was obtained and the growth inhibition of 50% (GI_50_), corresponding to the concentration of compound that inhibited 50% of the net cell growth, was determined as described elsewhere [16]. Doxorubicin used as a positive control, was tested in the same manner. Moreover the effect of the vehicle solvent (DMSO) on the growth of these cell lines was evaluated in all experiments by exposing untreated control cells to the maximum concentration (0.25%) of DMSO used in each assay.

## 4. Conclusions

In contrast to their dihydropyranoxanthone precursors **3-5**, the pyranoxanthones **6-8** did not exhibit growth inhibitory effect against the breast adenocarcinoma MCF-7 cells. On the other hand, *C*-prenylation of the inactive hydroxyxanthone **9**, led to prenylated derivatives **10** and **11** which exhibited moderate growth inhibitory activity against the MCF-cells. From these results, we can conclude that introduction of an unsaturation on the extra ring was not effective in improving the biological activity of these compounds. On the contrary, the introduction of the prenyl side chain on an appropriate position of the xanthonic scaffold was found to improve the antitumor activity of compounds **10** and **11**. The increase of the lipophilicity of the molecule and/or an extra molecular motif to interact with biological targets furnished by the prenyl group can be a key to explain the improvement this activity.
